# New Molecular and Organelle Alterations Linked to Down Syndrome Heart Disease

**DOI:** 10.3389/fgene.2021.792231

**Published:** 2022-01-18

**Authors:** Leslye Venegas-Zamora, Francisco Bravo-Acuña, Francisco Sigcho, Wileidy Gomez, José Bustamante-Salazar, Zully Pedrozo, Valentina Parra

**Affiliations:** ^1^ Advanced Center of Chronic Diseases (ACCDiS), Facultad de Ciencias Químicas y Farmacéuticas y Facultad de Medicina, Universidad de Chile, Santiago, Chile; ^2^ Departamento de Bioquímica y Biología Molecular, Facultad de Ciencias Químicas y Farmacéuticas, Universidad de Chile, Santiago, Chile; ^3^ Laboratory of Neuroprotection and Autophagy, Center for Integrative Biology, Faculty of Science, Universidad Mayor, Santiago, Chile; ^4^ Programa de Fisiología y Biofísica, ICBM, Facultad de Medicina, Universidad de Chile, Santiago, Chile; ^5^ Red para El Estudio de Enfermedades Cardiopulmonares de Alta Letalidad (REECPAL), Universidad de Chile, Santiago, Chile

**Keywords:** Down syndrome, chromosome 21, congenital heart defects, hypertrophy, oxidative stress, mitochondria, lysosome

## Abstract

Down syndrome (DS) is a genetic disorder caused by a trisomy of the human chromosome 21 (Hsa21). Overexpression of Hsa21 genes that encode proteins and non-coding RNAs (ncRNAs) can disrupt several cellular functions and biological processes, especially in the heart. Congenital heart defects (CHDs) are present in 45–50% of individuals with DS. Here, we describe the genetic background of this condition (Hsa21 and non-Hsa21 genes), including the role of ncRNAs, and the relevance of these new players in the study of the pathophysiology of DS heart diseases. Additionally, we discuss several distinct pathways in cardiomyocytes which help maintain a functional heart, but that might trigger hypertrophy and oxidative stress when altered. Moreover, we highlight the importance of investigating how mitochondrial and lysosomal dysfunction could eventually contribute to understanding impaired heart function and development in subjects with the Hsa21 trisomy. Altogether, this review focuses on the newest insights about the gene expression, molecular pathways, and organelle alterations involved in the cardiac phenotype of DS.

## 1 Introduction

Down syndrome (DS) is a genetic disorder mainly associated with intellectual disability, but which also involves a series of clinical conditions that affect multiple body systems, particularly the musculoskeletal, neurological, and cardiovascular systems (Antonarakis et al., 2020; Bull, 2020). DS is caused by a trisomy of the human chromosome 21 (Hsa21), due to nondisjunction or translocation of an additional copy of Hsa21 to another chromosome (Davisson et al., 1990). To a lesser extent, mosaicism, and partial trisomy 21 are other genetic diagnoses associated with the condition, but these are generally associated with fewer DS clinical features (Allen et al., 2009).

The physical features of DS were first described by John Langdon Down as common characteristics in some children with mental disability (Down, 1995). However, it was not until 1959 that Leujene *et al.* discovered that an extra copy of Hsa21 was the cause of the condition, after studying the somatic chromosomes of 9 children with DS (Lejeune et al., 1959). DS is currently considered a gene expression dysregulation disorder, due to the additional copy of Hsa21, producing effects not only on Hsa21 genes, but also causing genome-wide repercussions. Hence, the overexpression of Hsa21 genes encoding proteins and non-coding RNAs (ncRNAs) could eventually disrupt several cellular functions and biological processes, thus giving rise to the different clinical conditions of DS (Antonarakis, 2017). Some of the most common medical problems in subjects with DS are cognitive impairment, as well as early-onset of Alzheimer’s dementia, gastrointestinal malformations, congenital heart diseases, respiratory diseases, autoimmunity, thyroid dysfunction, and hematological disorders (Jensen and Bulova, 2014). Moreover, among the most notable DS clinical phenotypes are congenital heart defects (CHDs), in which case approximately 45–50% of DS individuals exhibit some forms of CHDs (Ferencz et al., 1989).

## 2 Cardiac Pathology in DS

CHDs are caused by alterations in the development and formation of the heart during the embryonic period. The consequences are defects in heart structure, which trigger problems in the pumping and circulation of blood (Freeman et al., 1998; Hawkins et al., 2011). The most frequent forms of CHDs in DS are atrioventricular septal defects (AVSDs), which contribute to 43% of CHD cases, and are almost exclusively present in DS. Other defects are ventricular septal defects (VSDs), atrial septal defects (ASDs), and the tetralogy of Fallot, corresponding to 32, 19, and 6%, respectively (Ferencz et al., 1989; Roizen and Patterson, 2003). Ethnic and sex differences in the prevalence of specific CHDs have also been underlined in DS (Versacci et al., 2018). Thus, the prevalence of the anatomic types of CHDs in DS is different in Asians and Native-American populations, compared to the Caucasian population; for instance, VSD is the most frequent CHD (43.16%) in the Asian population (Lo et al., 1989; Benhaourech et al., 2016). Evidently, these observations reflect the influence of other genetic variants and ethnic factors on the prevalence of CHD types in DS.

Interestingly, children with DS have a higher risk of developing pulmonary arterial hypertension (PAH) than the general population, partly due to CHDs. The increased pulmonary flow through the intracardiac shunt results in increased shear stress on pulmonary endothelial cells, which then leads to vascular remodeling and dysfunction (Hawkins et al., 2011). Despite specific treatments for PAH related to CHD, mortality still remains high, independently of the DS condition. Therefore, the best strategy for CHD PAH is prevention, through surgical repair of the heart defects.

Heart surgery represents a great advantage for the treatment of CHD in DS, and it should be performed as early as possible, conveniently within 3–6 months after birth, depending on the severity of the injury (Masuda et al., 2005). Non-operated cardiac injuries were the leading cause of death for subjects with DS, whose life expectancy averaged 12 years in the 1940s, increasing up to 30 years in the 1970s; meanwhile today, this parameter reaches an average of 60 years (Glasson et al., 2002; Presson et al., 2013). This increase in life expectancy has been directly related to early identification of heart defects and better results of corrective cardiac surgery (Masuda et al., 2005; Vis et al., 2009), also associated with a better general pediatric treatment.

The age at which surgical intervention is performed has decreased strongly to avoid the development of pulmonary hypertension; therefore, most children undergo surgery during the first months of life (Dennis et al., 2010). In children with CHDs, the indication for surgery is absolute, and in some situations, a second operation is necessary to finish repairing the heart valves and correct the associated insufficiencies (Dennis et al., 2010). However, these cardiac repairs do not always resolve the underlying PAH condition (Dennis et al., 2010; Hawkins et al., 2011). Therefore, it is crucial to find new strategies for the treatment of heart diseases, like PAH in DS.

## 3 Genetics of Heart Disease in DS

The nucleotide sequence of the long arm of Hsa21 (Hsa21q) was published in the year 2000 and since then, there has been a substantial progress in understanding the pathophysiology of the different phenotypic DS manifestations (Hattori et al., 2000). The Hsa21 is the smallest human chromosome and, according to the GENCODE project (GENCODE release 32), contains 233 protein-coding genes, 423 non-protein-coding genes (69 small, 330 long, and 24 non-miscellaneous coding genes), and 188 pseudogenes (Hattori et al., 2000; Antonarakis, 2017; Antonarakis et al., 2020). Multiple genes on Hsa21 and other genome sites, as well as epigenetic, environmental, and stochastics factors, are known to contribute to the clinical features of the syndrome. Hsa21 is one of the richest chromosomes in long non-coding RNAs (lncRNAs) and one of the poorest in micro-RNAs (miRNAs) and other ncRNAs (Antonarakis, 2017). Thus, understanding the genetic link between the susceptibility of subjects with DS to different morbidities constitutes an enormous challenge (Epstein et al., 1991).

Initially, reports described the existence of the Down syndrome critical region (DSCR). This region corresponds to the essential genes of Hsa21 responsible for the different DS phenotypes (Olson et al., 2004; Patterson, 2009). Although the current knowledge has demonstrated that key features of DS do not depend on the gene dosage effect of the DSCR (Amano et al., 2004; Olson et al., 2007), it is clear that the overexpression of the coding and non-coding Hsa21q genes affects the cellular processes and development of the individual. Thus, it might be a risk factor for developing some pathologies, such as CHD (Calcagni et al., 2017).

**FIGURE 1:**
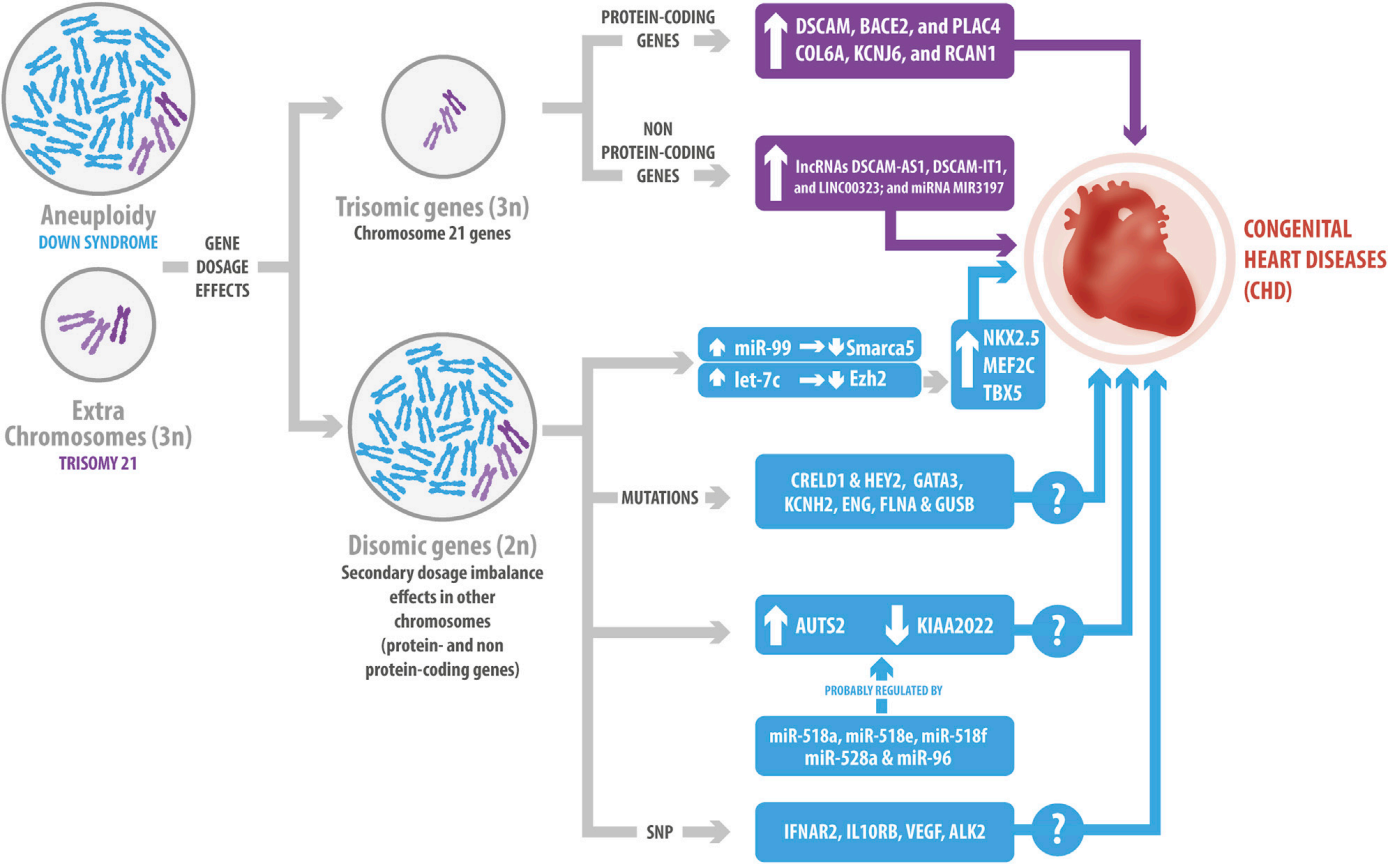
Genetic influence of Down Syndrome (DS) in congenital heart diseases (CHD). The gene dosage effects due to the overexpression of protein-coding and non-protein coding Hsa21genes have been related to CHD. Hsa21 protein-coding genes include *DSCAM*, *BACE2*, *PLAC4*, *COL6A*, *KCNJ6* and *RCAN1*. Hsa21 non-protein-coding genes include lncRNAs, such as *DSCAM-AS1*, *DSCAM-IT1* and *LINC00323*; and the *MIR3197* miRNA. The extra chromosome in DS changes the genomic context, generating a secondary dosage imbalance effect in other chromosomes. This leads to variations in the expression levels of non-Hsa21 genes, as well as mutations; such is the case of the changes observed for *CRELD1*, *HEY2*, *GATA3*, *KCNH2*, *ENG*, *FLNA* and *GUSB*, which might cause CHD. Additionally, the miR-99/let-7c cluster interacts with EZH2 and SMARCA5, regulating the expression of some transcriptional factors involved in cardiogenesis. Several miRNAs might have also a role in the development of CHD, such as miR-518a, miR-518e, miR-518f, miR-528a, and miR-96, which potentially co-regulate *AUST2* and *KIAA2022*. Finally, single nucleotide polymorphism has been associated with CHD, like the ones described for *IFNAR2*, *IL10RB*, *VEGF* and *ALK2.* However, several of the mechanisms underlying these effects still remain unclear.

Several investigations have studied Hsa21 gene overexpression to understand the relationship between DS and CHD (Figure 1). For instance, Pelleri *et al.* suggested a candidate DS-CHD region in Hsa21q22.2, with a length of 0.96 Mb, containing three coding genes involved (*DSCAM*, *BACE2,* and *PLAC4*) (Pelleri et al., 2017). *DSCAM* encodes for a cell adhesion protein that is expressed during cardiac development before endocardial cushion fusion. The gene imbalance due to trisomy 21 causes its overexpression before endocardial development in the fetal heart of mice and humans, starting at 12 weeks of development. DSCAM increases cell adhesion, thus affecting the attachment of endothelial cells, therefore inducing alterations in the cushion development that are present in CHD (Barlow et al., 2001). On the other hand, *BACE2* encodes for a protease that cleaves APP, contributing to the formation of amyloid-β plaque and the Alzheimer’s-related dementia observed in DS (Wang et al., 2019). PLAC4 is a placental protein whose mRNA expression has been studied as a non-invasive prenatal testing for DS, mainly focusing on the clinical value of its single polymorphism (SNP) rs8130833 (Wang et al., 2017). However, to date and despite all these data, there is no information about BACE2 and PLAC4 and their relationship with CHD. Moreover, other genes located in Hsa21, such as *COL6A*, *KCNJ6*, and *RCAN1* (Lange et al., 2004; Lignon et al., 2008; Grossman et al., 2011), have also been described as responsible for CHD (Figure 1). *COL6A* encodes for collagen VI. In this regard, fetal human heart staining has shown that collagen VI is present in the atrioventricular (AV) cushion, but its presence is higher in trisomy 21, thus suggesting its involvement in the development of the AV endocardial cushion (Gittenberger-de Groot et al., 2003). Similarly, Lignon *et al.* suggested that *KCNJ6*, which encodes a subunit of G protein-regulated K^+^ channels, can alter cardiac regulation and cause the DS arrhythmogenicity (Lignon et al., 2008). Furthermore, *RCAN1* (also known as *DSCR1*) participates in abnormal cardiac valvuloseptal formation, as well as the abnormal development of several organ systems affected in individuals with DS, including the heart, brain, eyes, ears, face, and limbs (Lange et al., 2004). Interestingly, restoration of RCAN1 to disomic levels (normal) did not rescue the cardiac development morphological abnormalities reported in the Ts16 mouse model for DS (Lange et al., 2005).

Recent case–control genome-wide association studies have identified other CHD risks loci located in the so-called “CHD critical region” on Hsa21 (Sailani et al., 2013). One of them maps near *RIPK4* and the second one includes the *ZBTB21* gene (Sailani et al., 2013)*.* The segment closest to *RIPK4* may contain regulatory elements for this gene. Consecuently, *RIPK4* overexpression has been observed in the heart of the Ts65Dn DS mouse model (Lyle et al., 2004). On the other hand, *ZBTB21* has been shown to interact with PPP2R2B, which, in *Drosophila*, regulates the WNT/β-catenin signaling pathway. This pathway has been described as necessary for cardiac differentiation of human embryonic stem cells (ESCs) (Calcagni et al., 2021). Additional analysis of copy number variants (CNV) has also shown that neither an individual chromosome 21 CNV nor any individual gene intersected by a CNV was associated with AVSD in DS (Rambo-Martin et al., 2018). In general, it has been suggested that CHD development is the result of a multifactorial model, with a cumulative effect in multiple risk alleles that exert minor or major contributions (Rambo-Martin et al., 2018; Calcagni et al., 2021). Altogether, these findings suggest that cardiac defects in DS are not the result of the overexpression of just one gene, but of a set of them.

As we previously mentioned, current knowledge indicates that the gene imbalance dosage of Hsa21 also affects gene expression in other locations (Li H et al., 2012; Alharbi et al., 2018). For instance, Li *et al.* identified two genes in DS mouse models: *CRELD1* and *HEY2* (Figure 1), which might be associated with AVSD. Both of them act as genetic modifiers, suggesting additive effects of dosage genes in the trisomy 21 condition, and that probably, there is a expression threshold that might result in CHD (Li YY et al., 2012; Rambo-Martin et al., 2018). In this regard, *CRELD1* encodes a cell surface protein that might participate in cell adhesion. Maslen *et al.* found two *CRELD1* missense mutations in children with DS and AVSD, implying its role in the pathogenesis of the disease (Maslen et al., 2006). Moreover, 22 variants of *CRELD1* have been identified in subjects with AVSD, but only one is present in the DS-AVSD group (Asim et al., 2018). HEY2 act as a transcriptional repressor during embryonic development. Thus, as reported, HEY2 limits the proliferation of cardiac progenitors in the heart of zebrafish (Gibb et al., 2018). Consequently, *HEY2* sequencing showed three nonsynonymous mutations in patients with AVSD, suggesting that *HEY2* could regulate ventricular septation (Reamon-Buettner and Borlak, 2006). Similarly, Alharbi *et al.* analyzed 240 blood samples from patients in order to identify genetic variants predictive of an increased risk factor for CHD in DS subjects. They described mutations in several genes present only in the CHD-DS group, such as *GATA3*, *KCNH2*, *ENG*, *FLNA*, and *GUSB*, which have been related to heart development and function (Alharbi et al., 2018). However, the corresponding mechanism has not been elucidated yet and requires further study.

Finally, as has been proposed, mutations and polymorphisms may contribute to the heterogeneity of DS phenotypes, such as the ones observed in the context of CHD. Balistreri *et al.* studied the relationship between SNPs in four interferon receptors (*IFNAR1*, *IFNAR2*, *IFNGR2*, *IL10RB*) and the *VEGFA* genes, and CHD in subjects with DS (Balistreri et al., 2020). The interferon receptor genes belong to a cluster located in a 3.7 Mb critical genomic region associated with DS heart defects in the Ts1Cje DS mouse model (Liu et al., 2014). Although *VEGFA* is located in another chromosome, its overexpression in mice embryos triggers severe heart development abnormalities (Miquerol et al., 2000). In this regard, Balisteri *et al.*, found that *IFNAR2* and *IL10RB* SNP were related with heart disorders in subjects with DS, whereas *VEGF* SNP were less prevalent in subjects with CHD (Balistreri et al., 2020). Similarly, Joziasse *et al.*, identified a SNP in the *ALK2* receptor*,* a member of the TGF-β superfamily, in a patient with DS and CHD, resulting in an H286D substitution. They demonstrated that this substitution reduced ALK2 function, which may contribute to CHD in the DS context (Joziasse et al., 2011). Interestingly, a previous investigation reported that ALK2 is expressed in the myocardium and endocardium of the developing heart. Furthermore, the endothelial-specific deletion of ALK2 shows atrioventricular septa and valve defects in mice (Wang et al., 2005). All of these results prove that Hsa21 overexpression may contribute to changes in genes from other chromosomes, causing different phenotypes associated with the CHD in the DS context.

### 3.1 Role of Non-Coding RNAs in DS CHD

ncRNAs are the most abundant transcriptional units of the genome, which can regulate gene expression through epigenetic mechanisms. The most studied ncRNAs are miRNAs and lncRNAs. Briefly, miRNAs are small RNAs of 21-25 nucleotides, while lncRNAs are transcripts with more than 200 nucleotides (Furuno et al., 2006; Antonarakis et al., 2020). Recent investigations have focused on the role of ncRNAs in the pathophysiology of DS and CHD. For instance, a genome-wide association study in DS subjects suggested that the non-Hsa21 FLJ33360 lncRNA could be involved in CHD by regulating its adjacent gene *MED10*, which is associated with cardiac defects (Ramachandran et al., 2015). *MED10* gene participates in heart valve formation in zebrafish by regulating the expression of the transcriptional regulator tbx2b in the AV myocardium (Just et al., 2016). In contrast, Pelleri *et al.* identified four ncRNAs (the *DSCAM-AS1*, *DSCAM-IT1*, and *LINC00323* lncRNAs; and the *MIR3197* miRNA) in their proposed DS-CHD region on Hsa21 (Pelleri et al., 2017) (Figure 1). *DSCAM-AS1* regulates proliferation, migration, and invasion of cancer cells by sponging miRNAs (Ji et al., 2019; Li B et al., 2020). *LINC00323* is a sensitive to hypoxia-lncRNA in endothelial cells, whose silencing results in angiogenic defects (Fiedler et al., 2015). Finally, miR-3197 has been proposed as a biomarker for diabetic retinopathy and hepatocellular carcinoma (Pascut et al., 2019; Ji et al., 2020). However, the regulatory effect of these ncRNAs in CHDs needs further investigation.

miRNAs also play an essential role in diverse cardiac cellular processes. Hsa21 contains 30 miRNAs that could potentially be overexpressed in DS (Amaral et al., 2018; Gomez et al., 2020). Several investigations have shown that miRNAs play a crucial role in heart development and diseases (Nishiguchi et al., 2015; Romaine et al., 2015; Zhou et al., 2018). Coppola *et al.* demonstrated that the miR-99a/let-7c cluster in Hsa21 is upregulated in human fetal hearts with DS. Overexpression of let-7c decreases its direct target EZH2, increasing transcription factors involved in cardiogenesis, such as NKX2.5, MEF2C, and TBX5. Therefore, let-7c regulates cardiomyogenesis positively. On the contrary, miR-99a represses cardiac differentiation, targeting Smarca5 in mouse ESCs (Figure 1). Moreover, they identified that the EZH2 and SMARCA5 miRNA targets decrease their expression in DS fetal hearts, suggesting that they might participate in CHD pathogenesis (Coppola et al., 2014). In another approach, an interaction network between predicted miRNAs and differentially expressed genes of DS subjects with AVSD indicated that *AUTS2* and *KIAA2022* are potentially co-regulated by five miRNAs (miR-518a, miR-518e, miR-518f, miR-528a, and miR-96) (Wang et al., 2016) (Figure 1). Interestingly, *AUST2* genomic alterations and mutations result in intellectual disabilities and microcephaly; while *KIAA2022* deficiency also produces intellectual disabilities, which have been related with impaired neurite outgrowth in rat hippocampal neurons (Van Maldergem et al., 2013; Beunders et al., 2016). However, to date there is no information about the role of *AUTS2* and *KIAA2022* in the heart.

Recently, a transcriptomic study showed that 447 transcripts were differentially expressed in subjects with DS, compared to controls (Salemi et al., 2021). Particularly, 203 transcripts were downregulated (151 protein-coding mRNAs, 45 lncRNAs, one microRNA, one mitochondrial tRNA, one ribozyme, and one small nuclear RNA) and 244 were overexpressed (210 protein-coding mRNAs and 34 lncRNAs). Prediction analyses indicated that the 89 deregulated lncRNA participated in developmental disorders, neurological diseases and cancer development in subjects with DS (Salemi et al., 2021). This investigation highlighted the importance of lncRNA in the different DS phenotypes, but whether they play a role in cardiac diseases is just starting to be investigated.

Given the mechanism of action of miRNAs, only one miRNA can virtually regulate the expression of several dozens of mRNAs. Moreover, lncRNAs can regulate gene expression by regulating the interaction between miRNAs and mRNAs (Mehler, 2008; Antonarakis et al., 2020). These mechanisms highlight the importance of applying not only genome and transcriptome-wide, but also systems biology approaches to integrate the contribution of miRNAs, lncRNAs, and other ncRNAs to the pathophysiology of DS heart diseases.

## 4 Molecular Cardiovascular Pathways in DS

As previously mentioned, one of the leading causes of death in DS subjects are CHDs; therefore, a vast majority of the investigations have focused on determining the molecular origin of these diseases. However, it is still unclear how the trisomy affects signaling pathways in cardiomyocytes related to the maintenance of a functional heart. Moreover, this information could also help understand the etiology of other heart conditions in non-DS populations.

### 4.1 Cardiac Hypertrophy Signaling in DS

**FIGURE 2:**
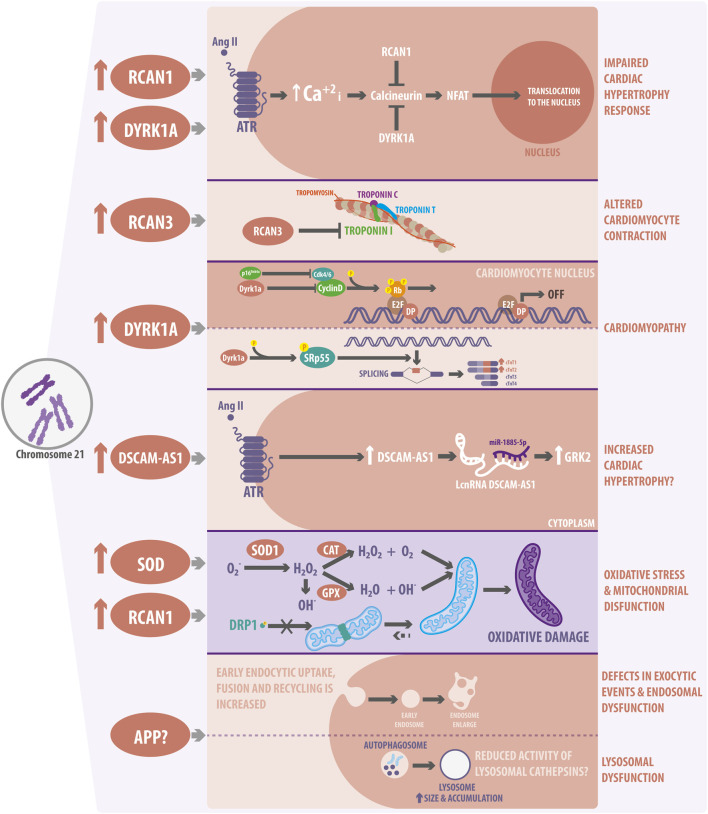
Cardiovascular molecular pathways and organelle alterations in Down Syndrome (DS). The overexpression of a few Hsa21 genes has been linked to alterations in cardiac function and development. RCAN1 and DYRK1A overexpression might impair cardiac hypertrophy response in DS by inhibiting CN activity and decreasing NFAT nuclear traslocation. RCAN3 can alter cardiomyocyte contraction by interacting with Troponin I. DYRK1A can also be involved in cardiomyopathies due to the altered regulation of cardiomyocyte proliferation and development. In this regard, DYRK1A interacts with D-cyclin family proteins and suppress Rb/E2F signaling. DYRK1A also modulates the splicing of cardiac troponin variants, promoting the inclusion of exon five into *TNNT2* through SRSF6 phosphorylation. DSCAM-AS1, which is overexpressed in DS, increases after Angiotensin II stimulation and interacts with miR-188-5p to stimulate GRK2, promoting an hypertrophic response. Moreover, overexpression of the DSCAM-AS1 lncRNA has been also related to cardiomyopathy. In terms of organelles, SOD1 overexpression increases ROS levels and oxidative stress, producing H_2_O_2_ accumulation, which finally leads to mitochondrial dysfunction. Additionally, RCAN1 regulates the connectivity of the mitochondrial network and its function, by regulating the phosphorylation of the mitochondrial fission protein, DRP1. Finally, trisomic cells show defects in the endocytic and endosomal pathways. They show decreased lysosomal activity and autophagy dysfunction related to APP overexpression. However, if APP is responsible for the lysosomes defects observed and described in cardiac DS cells is still not known.

Hypertrophy is an adaption mechanism in which cardiomyocyte size increases in response to an increased workload. There are two types of hypertrophy: physiological and pathological. The latter causes cardiac remodeling, which can progress to heart failure (Nakamura and Sadoshima, 2018). A master regulator of cardiac hypertrophy is NFAT, which stimulates the expression of hypertrophy-related genes after its translocation to the nucleus (Nakamura and Sadoshima, 2018). Increased intracellular calcium concentration activates calcineurin (CN), which dephosphorylates the NFAT transcription factor, promoting its nuclear translocation (Klee et al., 1998). Interestingly, *RCAN1*, localized on Hsa21 and increased in DS (Figure 2), inhibits CN activity and is highly expressed in the heart. As demonstrated, overexpression of RCAN1 can repress NFAT translocation to the nucleus (Fuentes et al., 2000). Moreover, NFAT transcriptional activity is decreased in the developing heart of Ts16 embryos, a murine DS model (Lange et al., 2005). Therefore, the evidence suggests that cardiac hypertrophy response to a stressful stimulus might be altered in DS cardiomyocytes. Furthermore, as described, another family member of the RCAN family is involved in DS cardiac alterations, specifically with cardiac contraction (Figure 2). According to Canaider *et al.*, RCAN3 (also known as DSCR1L2) interacts with cardiac troponin I (*TNNI3*) through a domain encoded in exon 2. TNNI3 is a subunit of the heart troponin complex and is essential for cardiac contraction (Canaider et al., 2006), indicating that this process could be altered in DS.

On the other hand, DYRK1A prevents cardiac hypertrophy *in vitro* by inhibiting myocardial NFAT signaling in cardiomyocytes (Grebe et al., 2011). DYRK1A is involved in different pathways in the heart, and its expression is also increased in DS (Figure 2). For instance, Hille *et al.* demonstrated that DYRK1A overexpression resulted in severe dilated cardiomyopathy due to impaired cardiomyocyte proliferation. DYRK1A interacts with D-type ciclins and reduces their protein levels, thus suppressing Rb/E2f signaling and impairing cell cycle progression in cardiomyocytes (Hille et al., 2016). Interestingly, another study also showed that phosphorylation of the SRSF6 splicing factor by DYRK1A modulates the splicing of cardiac troponin T (*TNNT2*) variants, promoting the inclusion of exon five into *TNNT2* in Ts65Dn mice, another murine DS model (Lu and Yin, 2016). Additionally, the evaluation of the DYRK1A-SRSF6-TNNT2 pathway in myocardial tissue from DS individuals showed elevated levels of phosphorylated SRSF6 and increased expression of fetal *TNNT2* variants (Quiñones-Lombraña and Blanco, 2019). These investigations revealed that cardiac contraction and proliferation might be dysregulated in DS, opening a completely new and relevant window of investigation. As previously mentioned, the overexpression of Hsa21-coding genes affects cardiac pathways. Therefore, non-coding genes could also dysregulate several cardiac signaling pathways in the same way. For instance, the DSCAM-AS1 lncRNA increases its expression in response to the pro-hypertrophy factor Angiotensin II in cardiomyocytes (Chen and Cai, 2020). Angiotensin II increases the levels of catecholamines, aldosterone, and vasopressin, as well as vasoconstriction and cardiac remodeling. Moreover, Chen *et al.* identified that DSCAM-AS1 sponged miR-188-5p to stimulate the GRK2 pro-hypertrophy gene (Chen and Cai, 2020). GRK2 is higly expressed in patients with heart failure and functions as an initiator of cardiac hypertrophy (Woodall et al., 2014). Remarkably, DSCAM-AS1 is overexpressed in DS, but its function has not been investigated in relation to DS hypertrophy (Pelleri et al., 2017) (Figure 2). Thus, it is essential to study the role of gene dosage imbalance in the cardiomyocyte signal transduction pathways related to DS.

Most of the investigations in DS-related diseases have been focused on CHD, probably because it is the most common heart disease in this condition (Ferencz et al., 1989). On the contrary, and despite the molecular pathways previously described; cardiac hypertrophy is rare in these subjects and its molecular mechanisms have not been fully studied (Assenza et al., 2007; Mahadevaiah et al., 2015). However, the study of cardiac hypertrophy in DS might shed new light on new treatment strategies, not only for DS individuals, but also for the general population.

### 4.2 Oxidative Stress in DS

The heart is one of the organs that consumes and produces more ATP; therefore, O_2_ homeostasis is important to maintain heart function. The heart needs to balance O_2_ supply to respond to changes in workload and pathological stresses, such as ischemia. Reactive oxygen species (ROS), such as O_2_^−^, ^−^OH, and H_2_O_2_, induce several signaling pathways essential for cardiac function (Wu et al., 2017). However, ROS imbalance may cause oxidative stress. Unfortunately, overproduction of ROS in DS is not normally compensated by antioxidant mechanisms (Schuchmann and Heinemann, 2000). Superoxide dismutase (Cu-Zn) type 1 (SOD1) catalyzes the dismutation of O_2_^−^ to molecular O_2_ and H_2_O_2_, which can be converted to water by catalase (CAT) and glutathione peroxidase (GPX), so this mechanism is considered the first line of antioxidant defense. SOD1 levels are 50% higher in DS cells and tissues, increasing H_2_O_2_ and ^−^OH production, which changes the relationship between SOD1 and its substrates: GPX and CAT (Groner et al., 1994; Zana et al., 2007) (Figure 2). In addition, increased activity of SOD1 dysregulates redox homeostasis, producing metabolic and gene expression alterations (Barone et al., 2018).

On the other hand, excessive ROS generation induces heart damage, especially during ischemia/reperfusion (I/R) injury. The major ROS increase is produced during the first minutes of myocardial reperfusion, especially through the reactivation of the electron transport chain in mitochondria (Zweier et al., 1987; Parra et al., 2018). Interestingly, DS subjects exhibit a reduced mortality rate from ischemic cardiovascular diseases: only ∼7%, compared, to 32% of the general population (Englund et al., 2013; Colvin and Yeager, 2017). A possible explanation for this is the overexpression of RCAN1 in DS. As reported, RCAN1-depleted cardiomyocytes are more sensitive to I/R injury, so RCAN1 overexpression could be a protective factor in the DS condition (Parra et al., 2018).

One of the primary sources of ROS production in cells are mitochondria, due to their inherent function of the electron transport chain. Interestingly, organelles such as mitochondria (Parra et al., 2018) and lysosomes (Jiang et al., 2019) are altered in DS; both of which will be discussed in more detail in the context of heart diseases in the final sections of this review.

### 4.3 Organelle Dysfunction in DS

Recent studies have highlighted the relationship between DS and organelle function, especially due to the chronic metabolic phenotype observed in DS individuals, such as obesity and type 2 diabetes (T2D) (Peiris et al., 2016). For example, T2D β-cells have shown increased ROS levels, mitochondrial dysfunction, and lower ATP levels; all phenotypes that have also been related with the reduced mitochondrial metabolism observed in DS cells (Helguera et al., 2013; Peiris et al., 2016). For instance, Peiris *et al.*, explored common genes between β-cells derived from DS and T2D pancreatic islets in order to understand the β-cell dysfunction described for DS individuals. They identified that RCAN1 is increased in T2D islets as in DS, and that its overexpression in mouse islets results in mitochondrial dysfunction and low ATP production. This lack of ATP then negatively affects both glucose-stimulated membrane depolarization and ATP-dependent insulin exocytosis (Peiris et al., 2016). Therefore, the study of DS at the organelle level has become more relevant to better understand the molecular mechanisms that lead to the metabolic phenotype occurring in the DS context.

#### 4.3.1 Mitochondrial Morphology and Function in DS

Mitochondria are highly dynamic organelles which, in response to stress, change their morphology to maintain their function. Alterations of mitochondrial morphology lead to impairments at the cardiovascular level, affecting processes such as cardiomyocyte differentiation, response to ischemic events, apoptosis, and autophagy (Ong and Hausenloy, 2010). Interestingly, fibroblast mitochondria from foetuses with DS show an increase in size, breaks in the inner membrane, and alterations in the mitochondrial cristae (Piccoli et al., 2013), evidencing altered mitochondrial morphology (Figure 2). Moreover, Piccoli *et al.* correlated these alterations with a decrease in the oxygen consumption rate (OCR) of DS fibroblasts as a readout of mitochondrial function. This OCR decrease was also accentuated in DS fibroblasts from foetuses with CHD (Piccoli et al., 2013), suggesting that mitochondria are an exciting target in the pathogenesis of CHD. In this line, Conti *et al.* demonstrated that genes encoding all five mitochondrial complexes and genes related to mitochondrial biogenesis were downregulated in hearts from DS subjects (Conti et al., 2007). Therefore, alterations in the mitochondrial complex genes might impair mitochondrial function and could explain the decreased OCR reported for some DS cells.

Mitochondrial morphology has also been studied in induced pluripotent stem cells (iPSC) from subjects with DS (Parra et al., 2018). Parra *et al.* showed that there is a reduced number of mitochondria per cell and an increase in their average volume, which are associated with a more interconnected mitochondrial network in trisomic iPSCs. However, this more connected mitochondrial network was associated with uncoupled oxygen consumption (Parra et al., 2018). RCAN1 depletion restores the mitochondrial network, together with oxygen consumption, indicating that the increased dosage of RCAN1 is responsible for the alteration of mitochondrial dynamics in the context of DS. Interestingly, overexpression of RCAN1 decreases the activation through dephosphorylation of the DRP1 mitochondrial fission protein, thus protecting cardiomyocytes from cell death induced by ischemia and reperfusion (Parra et al., 2018) (Figure 2).

Furthermore, increased oxidative stress is one of the main consequences of mitochondrial alterations and is a highly relevant feature of several cardiovascular diseases, such as hypertension and cardiac dysfunction (Ballinger, 2005). In this regard, reported evidence has indicated that trisomic fibroblasts present higher ROS production than disomic fibroblasts, and that redox imbalance is more significant in trisomic fibroblasts from foetuses with CHD (Piccoli et al., 2013). Consequently, Valenti *et al.*, has shown that the catalytic activity of the complex I decreases due to a decrease in cAMP-dependent phosphorylation of the complex. They also reported in trisomic fibroblasts a 3-fold increase of ROS levels, mainly originating in mitochondria (Valenti et al., 2011). Hence, oxidative stress and pathological remodeling of mitochondria can dysregulate several pathways and cellular communication with other subcellular organelles in the context of DS.

#### 4.3.2 Lysosome Function in the Context of DS

Lysosomes are organelles which contain various digestive enzymes responsible for breaking down multiple biomolecules and organelles to maintain cellular homeostasis. Novel descriptions have indicated that lysosome alterations are strongly related to the progress of cardiovascular diseases. For example, Danon disease, caused by a lysosomal storage disorder, is characterized by weakening of the heart muscle, causing cardiomyopathy, hypertrophy, and heart failure (Chi et al., 2020). Interestingly, iPSCs-derived cardiomyocytes from subjects with Danon disease show autophagosome accumulation due to blockade of autophagosome-lysosome fusion, generating mitochondrial damage and affecting the energy metabolism of these cells (Chi et al., 2019). Therefore, it is relevant to evaluate if lysosomal alterations contribute to the development of cardiovascular diseases in the context of DS.

Several investigations have described morphological alterations in lysosomes from DS patients, which contribute to some pathologies associated with this condition, but none in cardiovascular diseases. For instance, DS is the leading genetic risk factor for early-onset Alzheimer’s disease (AD) due to the increased dosage of APP, a Hsa21 gene. Thus, several investigations have focused on linking common features of DS and AD (Cataldo et al., 2008; Colacurcio et al., 2018; Jiang et al., 2019). Primary fibroblasts from DS subjects show impaired lysosomal degradation of autophagic and endocytic substrates, leading to an accumulation of enlarged autolysosomes/lysosomes. Jian *et al.*, demonstrated that overexpression of the APP gene impaired lysosomal acidification, causing autophagy dysregulation in cells from DS individuals and neurons of DS animal models. Alterations in lysosomes, endosomes, and autophagy were related to an increased β-cleaved carboxy-terminal fragment of APP (Jiang et al., 2019) (Figure 2). Another study showed that early endocytic uptake, fusion, and recycling were increased in DS fibroblasts, along with an increased number of enlarged endosomes (Cataldo et al., 2008). However, whether APP overexpression or another factor related with the impaired lysosome function observed in DS is also altered in DS cardiomyocytes and affects the development of CHD is still unknown.

Importantly, lysosomes are one of the main components of the autophagy pathway, which is crucial for maintaining cardiomyocyte morphology and function by regulating organelle turnover and cardiac remodeling (Wu et al., 2017). In this regard, mitophagy, the selective degradation of mitochondria by the autophagy machinery, is deficient in primary human fibroblasts derived from DS individuals (Bordi et al., 2019). Bordi *et al.*, reported that low Parkin and p62 levels delayed PINK1 activation, altering mitophagy in DS fibroblasts, thus leading to damaged mitochondria accumulation. Moreover, mTOR hyperactivity and reduced ATG proteins involved in autophagosome formation were the cause of this downregulated rate of mitophagy (Bordi et al., 2019). Similarly, a study described that impaired autophagy resulted in mitochondrial dysfunction and increased ROS in skeletal muscle and in pancreatic β-cells of mice (Wu et al., 2009), thereby highlighting the importance of the communication between mitochondria and lysosomes, as well as the repercussions of its alterations. Altogether, these findings suggest that the study of organelle dysfunction could contribute to a better understanding of heart function development and impairment in DS subjects. These results strongly highlight the relevance of investigating the underlying mechanisms of cardiac dysfunction in DS subjects at the organelle level. Subsequently, since organelle dysfunction causes several cardiac and chronic diseases, the study of how to reverse these dysfunctions is emerging as a promising therapeutic target in DS and several other related conditions.

## 5 Concluding Remarks

DS is a common chromosomal disease, found in 1–1.4 of 1,000 alive newborns (Roizen and Patterson, 2003; Bull, 2020). DS, like other diseases or anomalies, such as cancer, overweight or obesity, and HIV, among others, not only involves changes in the patient’s body, but also in their social and psychological environment. As it is well known, there is a lot of misinformation regarding subjects with DS, which also suffer stigmatization (Bastidas and Alcaraz, 2011; Azócar et al., 2017). Therefore, it is of uttermost relevance to generate and share knowledge about this syndrome. Currently, we do not have worldwide strong epidemiological data about the incidence and prevalence of DS. Moreover, the scenario is even more complex, since it is difficult to evaluate and compare in detail the differences between the health systems used by DS individuals and their families (Colvin and Yeager, 2017). Although DS individuals experience similar health conditions and use similar services, they are prone to experience multiple different conditions (Uppal et al., 2015; Colvin and Yeager, 2017).

Life expectancy for people with DS has increased to 60 years in recent decades, which is five times higher than the one they had in 1940 (Vis et al., 2009; Dennis et al., 2010). This increase in life expectancy is mainly due to the success of corrective heart surgeries for coronary heart disease in children with DS (Lange et al., 2007), which became possible thanks to advances and research in the area, and also to improved medical management and progress in educational, social, and financial support (Pfitzer et al., 2018). Research in DS patients has improved the life of hundreds of patients around the world, not only the ones affected with DS, but also of those who have benefited from the research generated from the study of this condition (Colvin and Yeager, 2017). We are sure that improving the lives of people with DS through new research that focuses in the impact of Hsa21 overexpression and its incidence in other coding and no-coding genes, as well as the study of new organelle relationships in the context of the DS condition will, in turn, improve the lives of people without DS worldwide. For instance, it is widely known and accepted that people with DS have lower rates of cardiac hypertrophy and I/R events, despite their elevated risk factors. In contrast, congenital heart disease is highly prevalent in DS (Ferencz et al., 1989; Assenza et al., 2007; Englund et al., 2013; Mahadevaiah et al., 2015; Colvin and Yeager, 2017). Thus, research into the mechanisms of resistance to development of coronary artery disease, I/R injury or cardiac hypertrophy in people with DS, will undoubtedly benefit the larger population.

Accordingly, RCAN1 overexpression in DS cardiac tissue could explain both, the decreased cardiac hypertrophy events and the diminished cardiac injury induced by I/R in DS patients. While RCAN1 is a physiological inhibitor of NFAT, one of the main pathways involved in cardiac hypertrophy (Nakamura and Sadoshima, 2018), this molecule also participates in cardiomyocyte protection against I/R injury, probably through mitochondrial dynamic regulation (Parra et al., 2018). Therefore, preservation of mitochondrial function could constitute the bridge between regulated ROS production and the reduced mortality rate observed in DS people after cardiac ischemic diseases. In parallel, chronic ROS overproduction in DS people could also be the key to explain the relation to I/R cardioprotection, rather than being associated with a harmfull signalling pathway. During preconditioning, increased ROS production is a crucial factor to prevent I/R damage (Hausenloy and Yellon, 2016). Even though this maneuver is acute in preclinical models, it is still unknown how a chronic and basal increase in redox tone can help prevent damage during cardiac ischemia and reperfusion processes. In this regard, RCAN1 is a promising therapeutic target that should continue to be studied in the heart tissue of people with DS. Moreover, other molecules that are overexpressed in DS patients and are also related with cardiac hypertrophy could unveil similar questions: Are the elevated expression levels of DYRK1A, RCAN3 and DSCAM-AS1 involved in the cardiac hypertrophic response prevention observed in DS individuals? What could we learn from here that can be useful for the treatment of cardiac hypertrophy and heart failure in the rest of the population? Indeed, the study of cardiac hypertrophy in DS might shed new light on new treatment strategies, not only limited to DS individuals.

Additionally, the interaction between mitochondrial degradation pathways and quality control, together with lysosome impairment, could be another question to aswer; as well as a pathway able to explain, at least part of the CHD observed in DS individuals. Thus, the decreased lysosome activity related with an altered autophagy process observed primarily in DS neurons (Jiang et al., 2019) could suggest a diminished or altered mitophagy that could, in turn, explain the mitochondrial dysfunction and increased oxidative ROS production observed in DS cardiac tissue. However, our knowledge about all these pathways in cardiac tissue during DS is still poor and increased research is required in this area to understand the observed phenomenons. For example, is APP involved in the endo-lysosomal defects reported in cardiomyocytes from DS donors? We currently do not know this seminal answer. Furthermore, the study of this phenomenon can shed new lights about the observed relationship between AD and heart failure (Cannon et al., 2017; Wolters et al., 2018; Li J et al., 2020) and probably, also help the search of new therapeutic targets for the treatment of both conditions.

In summary, with all these antecedents, we strongly advocate for more comparative studies focused on the cardiac diseases developed by subjects with and without DS, since these studies will lead to new strategies that will eventually prevent and treat diseases affecting millions of people worldwide.

## Glossary

AD: Alzheimer’s disease
ALK2: Activin receptor-like kinase-2
APP: Amyloid Precursor Protein
ASDs: Atrial septal defects
ATP: adenosine triphosphate
ATG: autophagy-related genes
AUST2: Autism susceptibility candidate 2 gene
AVSDs: Atrioventricular septal defects
BACE2: Beta-Secretase 2
CAT: Catalase
CHDs: Congenital heart defects
CNV: Copy number variants
COL6A: Collagen Type VI Alpha 1 Chain
CRELD1: Cysteine Rich With EGF Like Domains 1
DRP1: Dynamin-1-Like Protein
DS: Down Syndrome
DSCAM: Down Syndrome Cell Adhesion Molecule
DSCR1: Down Syndrome Critical Region-1
DYRK1A: Dual Specificity Tyrosine Phosphorylation Regulated Kinase 1A
E2f: E2F transcription factor 1
ENG: Endoglin, a TGF-beta binding protein of endothelial cells
ESCs: Embryonic stem cells
EZH2: Enhancer of Zeste 2 Polycomb Repressive Complex 2 Subunit
FLNA: Filamin A
GATA3: GATA binding protein 3
GRK2: G-protein-coupled receptor kinase 2
GPX: Glutathione Peroxidase
Hsa21: Human chromosome 21
HEY2: Hairy/enhancer-of-split related with YRPW motif protein 2
I/R: ischemia/reperfusion
IL10RB: Interleukin 10 receptor subunit beta
IFNAR: IFN alpha/beta receptor
IFNGR: Interferon-gamma receptor
iPSC: induced Pluripotent Stem Cells
KCNH2: Potassium Voltage-Gated Channel Subfamily H Member 2
KCNJ6: Potassium Inwardly Rectifying Channel Subfamily J Member 6
KIAA2022: NEXMIF-neurite extension and migration factor
lncRNAs: long non-coding RNAs
MEF2C: Myocyte enhancer factor 2C
miRNAs: micro-RNAs
mTOR: mammalian Target of Rapamycin
NFAT: Nuclear factor of activated T-cells
ncRNAs: non-coding RNAs
NKX2.5: NK2 Homeobox 5
OCR: Oxygen consumption rate
p62: SQSTM1 (Sequestosome 1)/ubiquitin-binding protein p62
PAH: Pulmonary arterial hypertension
PLAC4: Placenta-specific protein 4
PINK1: PTEN-induced kinase 1
PPP2R2B: Protein Phosphatase 2 Regulatory Subunit Bbeta
Rb: Retinoblastoma protein (pRb)
RCAN1: Regulator of Calcineurin 1
RIPK4: Receptor Interacting Serine/Threonine Kinase 4
ROS: Reactive oxygen species
SMARCA5: SJQ/SMB-related matrix-associated actin-dependent regulator of chromatin subfamily A member 5
SNP: Single polymorphism
SOD1: Superoxide dismutase (Cu-Zn) type 1
SRSF6: Serine/arginine-rich splicing factor 6
tbx2b: tbx2b T-box transcription factor 2b (zebrafish)
TBX5: T-box transcription factor 5
T2D: Type 2 diabetes
TNNI3: Troponin I3, cardiac type
TNNT2: Troponin T2, cardiac type
VEGFA: Vascular Endothelial Growth Factor A
VSDs: Ventricular septal defects
ZBTB21: Zinc Finger and BTB Domain Containing 21
